# Case Report: Characterization of a Novel NONO Intronic Mutation in a Fetus With X-Linked Syndromic Mental Retardation-34

**DOI:** 10.3389/fgene.2020.593688

**Published:** 2020-11-16

**Authors:** Hairui Sun, Lu Han, Xiaoshan Zhang, Xiaoyan Hao, Xiaoxue Zhou, Ruiqing Pan, Hongjia Zhang, Yihua He

**Affiliations:** ^1^Beijing Anzhen Hospital, Capital Medical University, Beijing, China; ^2^Key Laboratory of Medical Engineering for Cardiovascular Disease, Ministry of Education, Beijing, China; ^3^Beijing Key Laboratory of Maternal-Fetal Medicine and Fetal Heart Disease, Beijing Anzhen Hospital, Capital Medical University, Beijing, China; ^4^Beijing Chaoyang Hospital, Capital Medical University, Beijing, China; ^5^Affiliated Hospital of Innermongolia Medical University, Hohhot, China; ^6^Berry Genomics Co. Ltd., Beijing, China

**Keywords:** congenital heart disease, hypoplastic left heart syndrome, NONO (p54nrb), mini-gene splicing assay, intronic variant

## Abstract

**Background:**

The NONO gene is located on chromosome Xq13.1 and encodes a nuclear protein involved in RNA synthesis, transcriptional regulation, and DNA repair. Hemizygous variants in NONO have been reported to cause mental retardation, X-linked, syndromic 34 (MRXS34) in males. Due to the scarcity of clinical reports, the clinical characteristics and mutation spectrum of NONO-related disorder have not been entirely determined.

**Methods:**

We reported a fetus with hypoplastic left heart syndrome, performed a comprehensive genotyping examination, including copy-number variation sequencing and whole-exome sequencing, and screened for the genetic abnormality. We also conducted an *in vitro* mini-gene splicing assay to demonstrate the predicted deleterious effects of an intronic variant of NONO.

**Results:**

Exome sequencing identified a novel intronic variant (c.154 + 9A > G) in intron 4 of the NONO gene (NM_001145408.1). It was predicted to insert 4 bp of intron 4 into the mature mRNA. Minigene assay revealed that the c.154 + 9A > G variant caused the activation of the intronic cryptic splice site and 4 bp insertion (c.154_155ins GTGT) in mature mRNA. Literature review shows that cardiac phenotype, including left ventricular non-compaction cardiomyopathy and congenital heart disease, are consistent features of MRXS34.

**Conclusion:**

This study enlarges the mutation spectrum of NONO, further expands hypoplastic left heart syndrome to the phenotype of MRXS34 and points out the importance of intronic sequence analysis and the need for integrative functional studies in the interpretation of sequence variants.

## Introduction

The non-POU domain containing octamer-binding gene (NONO) is located on chromosome Xq13.1. It is a highly conserved member of the Drosophila behavior/human splicing protein family thought to be involved in RNA synthesis, transcriptional regulation, and DNA repair (Shav-Tal and Zipori, 2002). Hemizygous mutations in NONO have recently been reported to cause mental retardation, X-linked, syndromic 34 (MRXS34; OMIM: 300967) in several male patients (Mircsof et al., 2015; Reinstein et al., 2016; Scott et al., 2016; Carlston et al., 2019; Sewani et al., 2019; Sun et al., 2020b). The predominant presentation for these patients described so far is that of an X-linked recessive disorder characterized by delayed psychomotor development, intellectual disability with poor speech, dysmorphic facial features, and mild structural brain abnormalities, including thickening of the corpus callosum. Other findings may include a cardiac phenotype, including left ventricular non-compaction cardiomyopathy (LVNC) and congenital heart disease (CHD). However, due to the scarcity of clinical reports, the clinical characteristics and mutation spectrum of NONO-related disease are uncertain.

Hypoplastic left heart syndrome (HLHS) is a severe CHD involving hypoplasia of the left ventricle, aorta, and mitral valve. It is one of the most lethal CHD, accounts for approximately 4–8% of all CHD, and remains clinically challenging (Feinstein et al., 2012). HLHS is heritable and has been associated with various chromosomal abnormalities, including Turner and Jacobsen syndromes, and heterozygous mutations in NKX2.5, NOTCH1, MYH6, and GJA1 have been reported in a small number of cases (Feinstein et al., 2012; Tomita-Mitchell et al., 2016; Kim et al., 2020). However, the genetic basis of HLHS remains largely unknown.

Next-generation sequencing, especially whole-exome sequencing, has revolutionized clinical diagnostic testing and new disease gene and pathway recognition (Xue et al., 2015; Jin et al., 2017; Sun et al., 2020a). Approximately 85% of the disease-causing mutations can typically be found in the coding region or canonical splice sites (Choi et al., 2009). Increasing evidence indicates that intronic mutations that potentially affect splicing also play an important role in the etiology of human diseases (Vaz-Drago et al., 2017). Intronic mutations should be investigated when interpreting sequence variants in next-generation sequencing, especially when causative mutations cannot be identified in coding regions or canonical splice sites.

Here, we report a fetus with hypoplastic left heart syndrome (HLHS) carrying a novel intronic variant in NONO. Our case expands the mutation spectrum and the cardiovascular phenotypes associated with NONO mutations. We also reviewed the cardiac phenotype in patients with NONO mutations from the literature. Based on previously reported cases and our novel findings, we conclude that cardiac phenotype, including LVNC and CHD, are consistent features of MRXS34.

## Materials and Methods

The Institutional Review Board of the Medical Ethics Committee of Beijing Anzhen Hospital approved this study. The parents of the fetus provided their written informed consent to participate in this study.

### Fetal Ultrasound and Echocardiography Examination

The ultrasound examinations were performed using the General Electric Voluson E8 ultrasound system with transabdominal 2–4 MHz curvilinear transducers (GE Healthcare Ultrasound, Milwaukee, WI, United States). A complete fetal echocardiographic examination, including two-dimensional (2D), M-mode, color, and pulse Doppler echocardiography, was performed according to the American Society of Echocardiography guidelines and standards for performance of the fetal echocardiogram (Rychik et al., 2004).

### Copy Number Variation Sequencing and Whole-Exome Sequencing

Both copy-number variation sequencing and whole-exome sequencing were performed using methods described previously on genomic DNA from the deceased fetus and the parents (Sun et al., 2020a). Copy-number variation sequencing was used to identify aneuploidies and pathogenic copy number variants, while whole-exome sequencing was used to identify potential pathogenic sequence variants. Briefly, genomic DNA was extracted, hybridized and enriched for whole-exome sequencing. The captured libraries were sequenced using Illumina NovaSeq 6000 (Illumina, Inc., San Diego, CA, United States). Then, the sequencing data were aligned to the human reference genome (hg19/GRCh37) using BWA^1^ and PCR duplicates were removed by using Picard v1.57^2^. GATK^3^ was employed for variant calling. ANNOVAR^4^ was used for variant annotation and interpretation. We determined the frequency of each variant in the dbSNP150^5^, 1000 Genomes Project^6^ and gnomAD^7^ to remove common SNPs (minor allele frequency > 0.1%). Then, non-synonymous, splicing, frameshift and non-frameshift variants, as well as intronic variants within 20 base pairs of an exon, were prioritized for evaluation. SIFT^8^, PolyPhen-2^9^, MutationTaster^10^, and CADD^11^ were used to predict the pathogenicity of missense variants, while SpliceAI (Jaganathan et al., 2019), HSF^12^ and MatEntScan (Yeo and Burge, 2004) were used to evaluate the effects on splicing. Missense variants not presenting damaging results in any protein function prediction from SIFT, Polyphen2, MutationTaster and CADD were excluded. Intronic variants not presenting damaging results in any prediction from SpliceAI, HSF and MatEntScan were excluded. Pathogenicity of variants was determined according to current American College of Medical Genetics and Genomics guidelines that recommend classifying variants into five categories: pathogenic, likely pathogenic, uncertain significance, likely benign or benign (Richards et al., 2015).

### *In vitro* Mini-Gene Splicing Assay

#### Construction of Recombinant Plasmids

**FIGURE 1:**
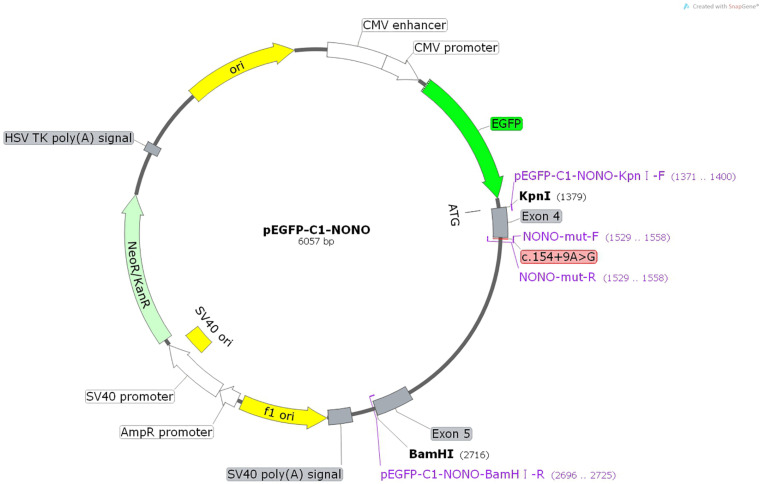
Structures and functional analysis of the splicing vector pEGFP-C1 and mini-gene NONO-wt/NONO-mut (c.154 + 9A > G).

**FIGURE 2:**
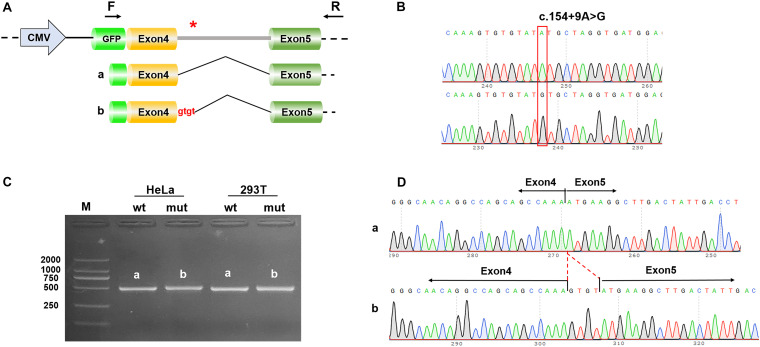
Results of the mini-gene splicing assay. **(A)** Schematic diagram of mini-gene construction and the c.154 + 9A > G variant related abnormal splicing. The asterisk indicates the location of the c.154 + 9A > G variant. **(B)** Sequencing results of the target fragment of NONO-wt (upper panel) and NONO-mut (lower panel) mini-gene. **(C)** Gel electrophoresis of RT-PCR products: the band of the mutant was bigger than the wild-type. **(D)** Mini-gene product sequencing results: (a) The wild-type mini-gene (NONO-wt) formed normal mRNA composed of exons 4 and 5; (b) The mutant mini-gene (NONO-mut) caused a splicing abnormality, resulting in the retention of the 4 bp in intron 4.

The constructs were made by three consecutive rounds of PCR: the first round was performed using genomic DNA (a total of two sets of DNA) as a template, and NONO-6070-F and NONO-9726-R as primers; the secondary round was performed using the first round of PCR products as a template, and NONO-6556-F and NONO-9241-R as primers; the third round was performed using PCR product of the second round as a template, and pEGFP-C1-NONO-KpnI-F and pEGFP-C1-NONO-BamHI-R as primers (Supplementary Table 1). The electrophoresis and gel recovery were performed for the final PCR products. Both NONO-wt and NONO-mut (c.154 + 9A > G) PCR products contained the entire sequence of exon 4 to exon 5, and the amplified length was 1,335 bp. PCR products of NONO-wt and NONO-mut (c.154 + 9A > G) were purified and inserted into the eukaryotic expression vector pEGFP-C1 using Kpn/BamHI to construct two sets of plasmids: pEGFP-C1-NONO-wt and pEGFP-C1-NONO-mut (Figures 1, 2A). The recombinant plasmids were digested with *Kpn*I and *Bam*HI, and verified by gene sequencing (Figure 2B).

#### Transfection of Eukaryotic Cells

Inoculate 5 × 105 cells (HEK-293T and Hela) with 1 ml medium (DMEM, 10% fetal bovine serum, no antibiotics) per well in a 12-well plate, and when the cell density reaches 90%, dilute in Opti-MEM (Gibco, United States) 1 μg plasmid and 3 μl liposome transfection reagent (Yeasen Biotech Company Limited, Shanghai, China), according to the instructions of liposome transfection reagent after incubation is completed for transfection. Collect cells for analysis 48 h after transfection.

#### Real Time-Polymerase Chain Reaction

The total RNA was extracted from 293T, and HeLa cells using a RNA extraction kit (TaKaRa) by Trizol method, and the cDNA was reverse transcribed using a reverse transcription kit (YESEN) according to the manufacturer’s instructions. The concentration and purity of the extracted RNA were determined by UV spectrophotometry. PCR products were identified by 2% agarose gel electrophoresis and verified by sequencing.

## Results

### Clinical Phenotypes

**FIGURE 3:**
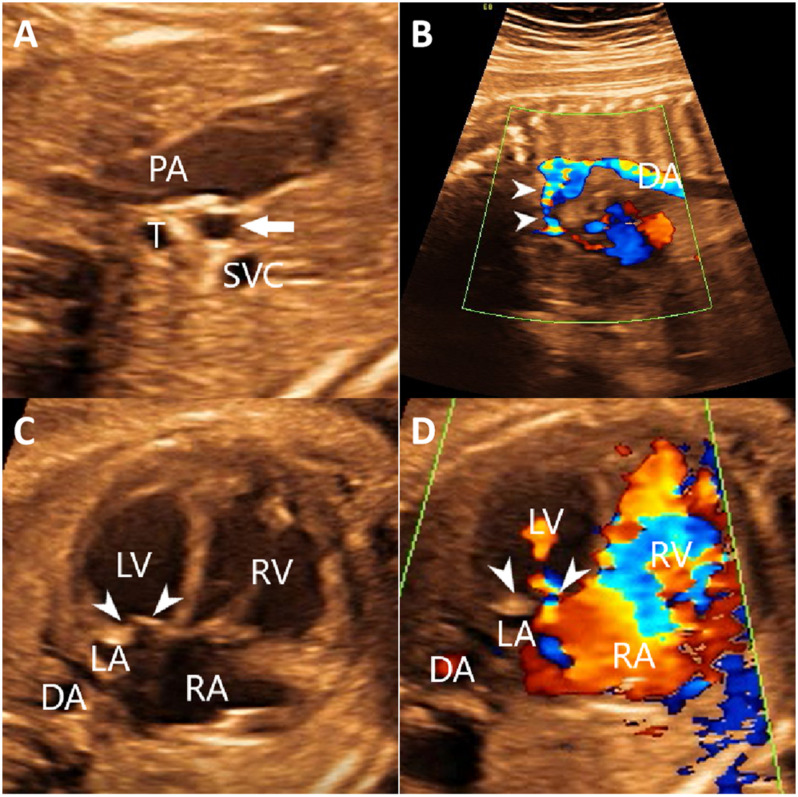
The echocardiography of the fetus identified aorta hypoplasia **(A,B)** hypoplastic left ventricle and Mitral valve dysplasia **(C,D)**. **(A)** Two-dimensional echocardiography showed the thin, small ascending aorta (arrow). **(B)** Color Doppler echocardiography showed the narrowed aortic arch (arrows) and retrograde flow in the aortic arch. **(C)** Two-dimensional echocardiography showed that the thickened mitral leaflets with enhanced echo. The ratio of the right heart to the left heart was increased. **(D)** Color Doppler showed a limited mitral valve (arrows) opening, reduced forward flow, and reduced left ventricular filling. DA, descending aorta; LA, left atrium; LV, left ventricle; PA, pulmonary artery; RA, right atrium; RV, right ventricle; SVC, superior vena cava; T, trachea.

A 28-year-old primigravida was referred at 26 weeks’ gestation for prenatal echocardiography. Both the woman and her partner were healthy, with no significant family history, and did not take any medication. She and her partner were non-consanguineous. Detailed fetal echocardiographic examination revealed hypoplastic left ventricle, Mitral valve dysplasia, and aorta hypoplasia (Figure 3). The echocardiographic findings were consistent with a prenatal ultrasound diagnosis of HLHS. Ultrasound screening in the first and second trimesters detected no extra-cardiac abnormalities. After detailed counseling, the couple decided to terminate the pregnancy and undergo genetic testing but refused to perform an autopsy on the fetus.

### Molecular Findings

**FIGURE 4:**
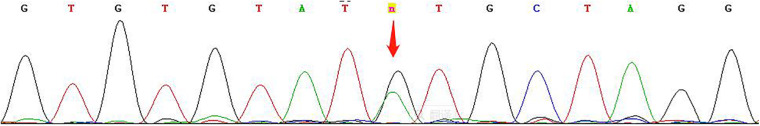
Sanger sequencing shows that the mutation is heterozygous in the mother.

Trio copy number variation sequencing and whole-exome sequencing were performed on DNA extracted from samples of the fetus and the parents to determine the underlying genetic cause of the fetal cardiac phenotype. Copy-number variation sequencing identified no chromosomal abnormalities. The mean sequencing coverage on target regions of whole-exome sequencing was 105-fold, providing enough data to obtain 96.21% at 20 × coverages of 64 Mb targeted exome of the human genome (hg19). Based on the aligned reads, 81,885 initial variants were identified. The filtering cascades for WES data are listed in Supplementary Table 2. After five filters of the variants data for WES data, 67 variants were kept (Supplementary Table 3). Finally, we identified a maternally inherited hemizygous variant, c.154 + 9A > G, in intron 4 of the NONO gene (NM_001145408.1). The next-generation sequencing data revealed the presence of this mutation in 93 out of 93 (93:0) reads in the proband, and 96:73 and 0:61 of the mutation vs. the wild-type reads in the mother and the father, respectively. Subsequent Sanger sequencing confirmed the heterozygous mutation in the mother (Figure 4). Besides finding the NONO mutation presented above, we also identified a paternal inherited, rare and putatively damaging variant in the HLHS-associated gene MYH6 (NM_002471.3:c.718G > A, p.Asp240Asn).

### *In silico* Splicing Analysis

Computational predictions conducted using SpliceAI, HSF and MatEntScan revealed that the intronic c.154 + 9A > G variant might influence the splicing process by differentially affecting canonical vs. cryptic splice site utilization. The newly detected variant was predicted by HSF to insert 4 bp of intron 4 into the mature mRNA (Figure 2A). This variant should give rise to frameshift and a premature termination codon (p.Asn52Serfs^∗^3).

### Results of the Mini-Gene Splicing Assay

To prove that the c.154 + 9A > G variant caused the 4 bp insertion, we then conducted the mini-gene splicing experiment. The mini-gene splicing products were analyzed by PCR amplification with plasmid-specific primers and visualized with polyacrylamide gel electrophoresis.

RT-PCR analysis using wild-type and mutant minigene constructs revealed that, compared with the wild-type construct, the PCR products generated by the mutant c.154 + 9A > G construct were slightly larger (Figure 2C). The amplicons confirmed by Sanger sequencing showed that the cDNA fragment obtained from the c.154 + 9A > G plasmid contained an additional 4 bp in intron 4 to exon 4 (c.154_155ins GTGT) (Figure 2D). This result is consistent with the *in silico* analysis result.

## Discussion

This study describes a fetus with HLHS that carried a novel intronic variant in the NONO gene. Minigene assay revealed that the variant caused the activation of a cryptic splice site and 4 bp insertion in mature mRNA. Our case enlarges the mutation spectrum of NONO and expands hypoplastic left heart syndrome to the phenotype of NONO related disorder.

This novel intronic variant in the NONO gene (c.154 + 9A > G) has previously not been reported as pathogenic or benign and has not been observed in the general population (dbSNP150, 1000 Genomes Project, gnomAD). It showed a deleterious effect by multiple *in silico* algorithms. Furthermore, *in vitro* experiment of mini-gene splicing assay verified that the variant alters splicing, leading to frameshift and a predicted premature termination codon. It is expected to result in either an abnormal truncated protein product or loss of protein from this allele through nonsense-mediated mRNA decay. In addition, the majority of reported patients with NONO mutations presented with CHD (Mircsof et al., 2015; Reinstein et al., 2016; Scott et al., 2016; Carlston et al., 2019; Sewani et al., 2019; Sun et al., 2020b) suggesting the association between NONO deficiency and CHD. In conclusion, we classified this NONO mutation as pathogenic according to the American College of Medical Genetics and Genomics guidelines (Richards et al., 2015). In addition to the NONO variant inherited from the mother, a rare and putatively damaging MYH6 variant inherited from the father was also identified. Recently, rare variants in MYH6 have been observed in 10.5% of HLHS patients, and sarcomere disorganization and functional defects have been demonstrated in tissue and induced pluripotent stem cell-derived cardiomyocytes from patients with MYH6 mutations (Tomita-Mitchell et al., 2016; Kim et al., 2020). Both parents are asymptomatic, and the NONO mutation and the MYH6 mutation are inherited from the mother and father, respectively. Generally, for X-linked recessive genetic diseases (NONO mutation), female heterozygotes (the mother in this report) have no phenotype and only have phenotypes when they are homozygous; on the contrary, for autosomal dominant genetic diseases (MYH6 mutation), heterozygotes (the father) should have phenotypes, of course, there are also incomplete penetrance phenomena. However, autosomal dominant with low penetrance is not an expected modality of CHD genetics. Therefore, we believe that the NONO mutation is the major genetic factor causing the fetal phenotype, and the MYH6 mutation may be a modifier that may affect the fetal HLHS. In this work, we highlighted the importance of including extended intronic regions in the analysis of WES results during variant prioritization and post-analytical phase for clinical purposes. This work also points out that functional investigations can be a powerful tool in support of pathogenicity of mutations.

Interestingly, while structural cardiac defects and LVNC have been reported in MRXS34 (Reinstein et al., 2016; Scott et al., 2016; Carlston et al., 2019; Sewani et al., 2019; Sun et al., 2020b), HLHS has not been previously associated with NONO-related disorders. Although this cardiac abnormality might be coincidentally present in the fetus herein described, previous frequent reports of other structural cardiac abnormalities suggest a possible involvement of the NONO defect. Recent experiment showed that NONO deficiency was associated with developing heart defects in mice (Xu et al., 2019), further suggesting the role NONO in cardiac development. Further research is needed to investigate the underlying mechanism of NONO deficiency causing CHD (including HLHS) are needed.

MRXS34 is a very rare X-linked recessive disorder, the incidence of which is unknown. A detailed literature review associated with the data from our subject showed that all of the patients are male. Prenatal abnormalities were seen in more than half of the patients (10/17), including cardiac abnormalities (LVNC and CHD) (6/17), polyhydramnios (2/17), intrauterine growth retardation (2/17), macrocephaly (1/17), and short limbs (1/17) (Table 1).

**TABLE 1:** Cardiac phenotype and prenatal findings of individuals with NONO mutations.

References, subject identifier	Variant (NM_001145408.2)	LVNC (age at diagnosis)	Congenital heart disease	Prenatal findings
Mircsof et al. (2015), MCCID1	c.1131G > A, p.Ala377Ala	NR	NR	NR
Mircsof et al. (2015), MCCID2	c.1394dupC, p.Asn466Lysfs*13	NR	NR	Hydramnios
Mircsof et al. (2015), DDD4K.0140	c.1093C > T, p.Arg365*	NR	NR	Intrauterine growth retardation
Reinstein et al. (2016)	c.1171 + 1G > T	YES (8 m)	NR	NR
Scott et al. (2016), Subject 1	c.1093C > T, p.Arg365*	YES (1 m)	Right ventricular hypertrophy, ASD, VSD, PDA	NR
Scott et al. (2016), Subject 2	c.1394dupC, p.Asn466Lysfs*13	YES (at birth)	ASD, VSD, PDA	NR
Scott et al. (2016), Subject 3	Exons 1–6 deletion	YES (1 m)	PFO	NR
Carlston et al. (2019)	c.154 + 5_154 + 6delGT, p.Asn52Serfs*6	YES (2 m)	EA, PFO	Intrauterine growth retardation
Sewani et al. (2019), S1	c.550C > T, p.Arg184*	YES (ab birth)	ASD, VSD, PDA, aortic dilatation	NR
Sewani et al. (2019), S2	c.1171 + 1G > A	YES* (4 y)	NR	Polyhydramnios, short limbs, brain abnormality, macrocephaly
Sewani et al. (2019), S3	c.457C > T, p.Arg153*	NR	Cardiomegaly, PS	Abortion at 16 weeks.
Sun et al. (2020b), A1	c.246_249del, p.Pro83Thrfs*7	YES (prenatal)	EA, PS, VSD, pericardial effusion	Cardiac abnormalities
Sun et al. (2020b), A2	c.246_249del, p.Pro83Thrfs*7	YES (prenatal)	Pulmonary atresia, VSD, aorta astride, right aortic arch, pulmonary dysplasia, transposition of the aorta, PLSVA	Cardiac abnormalities
Sun et al. (2020b), A3	c.246_249del, p.Pro83Thrfs*7	YES (prenatal)	PS, VSD, right ventricular diverticulum, PLSVA	Cardiac abnormalities
Sun et al. (2020b), B2	c.471del, p.Gln157Hisfs*18	YES (prenatal)	EA, PS, ASD, variation of branch of aortic arch	Cardiac abnormalities
Sun et al. (2020b), B3	c.471del, p.Gln157Hisfs*18	YES (prenatal)	EA, PS, VSD	Cardiac abnormalities
This study	c.154 + 9A > G, p.Asn52Serfs*3	NR	Hypoplastic left ventricle, Mitral valve dysplasia, aorta hypoplasia	Cardiac abnormalities

ASD, atrial septal defect; EA, Ebstein’s anomaly; LVNC, left ventricular non-compaction cardiomyopathy; NR, not reported; PDA, patent ductus arteriosus; PFO, patent foramen ovale; PLSVA, persistent left superior vena cava; PS, Pulmonary stenosis; VSD, ventricular septal defect. *Mildly increased concentric left ventricular wall thickness with some features of LVNC.

A total of 17 individuals with NONO mutations have been described, including the fetus reported herein. 14 (82%) of these patients present with cardiac phenotype, including LVNC and CHD (Table 1). LVNC is the most common cardiac phenotype, manifested in 12 (71%) of 17 patients with NONO mutations. Interestingly, of the 12 LVNC patients with NONO mutation, 10 (83%) were accompanied by CHD. The incidence of CHD in LVNC patients caused by NONO mutations seems to be significantly higher than its incidence in overall LVNC patients (Oechslin and Jenni, 2018; Richard et al., 2018; van Waning et al., 2018, 2019a,b; Kayvanpour et al., 2019). In addition, LVNC is diagnosed early in all patients with NONO mutations: five in the prenatal period (Sun et al., 2020b), four in the neonatal period (Scott et al., 2016; Sewani et al., 2019), two in infancy (Reinstein et al., 2016; Carlston et al., 2019), and one at the age of 4 (Sewani et al., 2019), indicating that LVNC is an early diagnostic clue for MRXS34. The severity of cardiac symptoms in these patients varies, ranging from requiring no medical intervention to requiring cardiac transplantation. These observations suggest that the NONO mutation may predispose males to the development of LVNC and CHD with high but incomplete penetrance and other unknown factors affecting their penetrance and expressivity. Further work will hopefully elucidate the pathophysiological mechanism of the association between the dysfunction of NONO and LVNC.

CHD is also frequent in patients with NONO mutations, characterized by septal defects and right-sided lesions (Table 1). The prevalence of CHD is 71% (12/17), which included 59% (*n* = 10) with septal defects, including ventricular septal defect, atrial septal defect, and patent foramen ovale and 41% (*n* = 7) with right-sided lesions including pulmonary stenosis/atresia and Ebstein’s anomaly.

### Study Limitations

Although this fetus is most likely to be MRXS34 according to its NONO mutation, we cannot distinguish whether its HLHS is isolated or part of MRXS34. Because, first, the neurodevelopmental status cannot be evaluated during the fetal period, and the pregnancy has been terminated, so we cannot determine whether the fetus would have the most common neurodevelopmental abnormalities of MRXS34 if it was born. Second, subtle dysmorphic features of MRXS34 cannot be detected on ultrasound. Finally, a fetal autopsy was not performed without parental consent to obtain additional clinical information.

## Conclusion

In conclusion, this study enlarges the mutation spectrum of NONO, further expands the phenotype to include HLHS, and points out the importance of intronic sequence analysis and the need for integrative functional studies in the interpretation of sequence variants. By combining our case and literature review, we conclude that cardiac phenotypes, including LVNC and CHD, are consistent features of NONO-related disorder. Further investigations into the pathophysiological mechanism associated with NONO variants and genotype-phenotype correlations are needed.

## Data Availability Statement

All datasets generated for this study are included in the article/Supplementary Material, further inquiries can be directed to the corresponding authors.

## Ethics Statement

The studies involving human participants were reviewed and approved by the Institutional Review Board of the Medical Ethics Committee of Beijing Anzhen Hospital. The patients/participants provided their written informed consent to participate in this study. Written informed consent was obtained from the minor(s)’ legal guardian/next of kin for the publication of any potentially identifiable images or data included in this article.

## Author Contributions

HZ, HS, and YH designed the study. XSZ, XH, and XXZ collected the clinical data and samples from the family. LH and RP completed the experiments. HS and LH analyzed and interpreted the data, and wrote the manuscript. All authors read and approved the final manuscript.

## Conflict of Interest

RP was employed by the company Berry Genomics Co. Ltd. Beijing, China. The remaining authors declare that the research was conducted in the absence of any commercial or financial relationships that could be construed as a potential conflict of interest.

## Footnotes

1 http://bio-bwa.sourceforge.net/

2 http://picard.sourceforge.net/

3 https://software.broadinstitute.org/gatk/

4 http://wannovar.wglab.org/

5 https://www.ncbi.nlm.nih.gov/snp/

6 http://www.internationalgenome.org/

7 https://gnomad.broadinstitute.org/

8 http://sift.jcvi.org

9 http://genetics.bwh.harvard.edu/pph2

10 http://www.mutationtaster.org

11 http://cadd.gs.washington.edu

12 http://www.umd.be/HSF3/

## Abbreviations

CHD: congenital heart disease
HLHS: hypoplastic left heart syndrome
LVNC: left ventricular non-compaction cardiomyopathy
MRXS34: mental retardation, X-linked, syndromic 34.

## References

[B1] Carlston C. M. Bleyl S. B. Andrews A. Meyers L. Brown S. Bayrak-Toydemir P. (2019). Expanding the genetic and clinical spectrum of the NONO-associated X-linked intellectual disability syndrome. *Am. J. Med. Genet. A* 179 792–796. 10.1002/ajmg.a.61091 30773818

[B2] Choi M. Scholl U. I. Ji W. Liu T. Tikhonova I. R. Zumbo P. (2009). Genetic diagnosis by whole exome capture and massively parallel DNA sequencing. *Proc. Natl. Acad. Sci. U.S.A.* 106 19096–19101. 10.1073/pnas.0910672106 19861545 PMC2768590

[B3] Feinstein J. A. Benson D. W. Dubin A. M. Cohen M. S. Maxey D. M. Mahle W. T. (2012). Hypoplastic left heart syndrome: current considerations and expectations. *J. Am. Coll. Cardiol.* 59 S1–S42. 10.1016/j.jacc.2011.09.022 22192720 PMC6110391

[B4] Jaganathan K. Kyriazopoulou Panagiotopoulou S. Mcrae J. F. Darbandi S. F. Knowles D. Li Y. I. (2019). Predicting splicing from primary sequence with deep learning. *Cell (Cambridge)* 176 535–548. 10.1016/j.cell.2018.12.015 30661751

[B5] Jin S. C. Homsy J. Zaidi S. Lu Q. Morton S. DePalma S. R. (2017). Contribution of rare inherited and de novo variants in 2,871 congenital heart disease probands. *Nat. Genet.* 49 1593–1601. 10.1038/ng.3970 28991257 PMC5675000

[B6] Kayvanpour E. Sedaghat-Hamedani F. Gi W. Tugrul O. F. Amr A. Haas J. (2019). Clinical and genetic insights into non-compaction: a meta-analysis and systematic review on 7598 individuals. *Clin. Res. Cardiol.* 108 1297–1308. 10.1007/s00392-019-01465-3 30980206

[B7] Kim M. Fleres B. Lovett J. Anfinson M. Samudrala S. S. K. Kelly L. J. (2020). Contractility of induced pluripotent stem cell-cardiomyocytes with an MYH6 head domain variant associated with hypoplastic left heart syndrome. *Front. Cell Dev. Biol.* 8:440 10.3389/fcell.2020.00440 PMC7324479 32656206

[B8] Mircsof D. Langouet M. Rio M. Moutton S. Siquier-Pernet K. Bole-Feysot C. (2015). Mutations in NONO lead to syndromic intellectual disability and inhibitory synaptic defects. *Nat. Neurosci.* 18 1731–1736. 10.1038/nn.4169 26571461 PMC5392243

[B9] Oechslin E. Jenni R. (2018). Left ventricular noncompaction. *J. Am. Coll. Cardiol.* 71 723–726. 10.1016/j.jacc.2017.12.031 29447732

[B10] Reinstein E. Tzur S. Cohen R. Bormans C. Behar D. M. (2016). Intellectual disability and non-compaction cardiomyopathy with a de novo NONO mutation identified by exome sequencing. *Eur. J. Hum. Genet.* 24 1635–1638. 10.1038/ejhg.2016.72 27329731 PMC5110068

[B11] Richard P. Ader F. Roux M. Donal E. Eicher J. C. Aoutil N. (2018). Targeted panel sequencing in adult patients with left ventricular non−compaction reveals a large genetic heterogeneity. *Clin. Genet.* 95 356–367. 10.1111/cge.13484 30471092

[B12] Richards S. Aziz N. Bale S. Bick D. Das S. Gastier-Foster J. (2015). Standards and guidelines for the interpretation of sequence variants: a joint consensus recommendation of the American College of Medical Genetics and Genomics and the Association for Molecular Pathology. *Genet. Med.* 17 405–423. 10.1038/gim.2015.30 25741868 PMC4544753

[B13] Rychik J. Ayres N. Cuneo B. Gotteiner N. Hornberger L. Spevak P. J. (2004). American Society of Echocardiography guidelines and standards for performance of the fetal echocardiogram. *J. Am. Soc. Echocardiogr.* 17 803–810. 10.1016/j.echo.2004.04.011 15220910

[B14] Scott D. A. Hernandez-Garcia A. Azamian M. S. Jordan V. K. Kim B. J. Starkovich M. (2016). Congenital heart defects and left ventricular non-compaction in males with loss-of-function variants in NONO. *J. Med. Genet.* 54 47–53. 10.1136/jmedgenet-2016-104039 27550220

[B15] Sewani M. Nugent K. Blackburn P. R. Tarnowski J. M. Hernandez−Garcia A. Amiel J. (2019). Further delineation of the phenotypic spectrum associated with hemizygous loss-of-function variants in NONO. *Am. J. Med. Genet. A* 182 652–658. 10.1002/ajmg.a.61466 31883306

[B16] Shav-Tal Y. Zipori D. (2002). PSF and p54(nrb)/NonO–multi-functional nuclear proteins. *FEBS Lett.* 531 109–114. 10.1016/S0014-5793(02)03447-6 12417296

[B17] Sun H. Yi T. Hao X. Yan H. Wang J. Li Q. (2020a). Contribution of single−gene defects to congenital cardiac left−sided lesions in the prenatal setting. *Ultrasound Obst. Gyn.* 56 225–232. 10.1002/uog.21883 31633846

[B18] Sun H. Zhou X. Hao X. Zhang Y. Zhang H. He Y. (2020b). Characteristics of cardiac phenotype in prenatal familial cases with NONO mutations. *Circ. Genom. Precis. Med.* 13:e2847 10.1161/CIRCGEN.119.002847 32397791

[B19] Tomita-Mitchell A. Stamm K. D. Mahnke D. K. Kim M. S. Hidestrand P. M. Liang H. L. (2016). Impact of MYH6 variants in hypoplastic left heart syndrome. *Physiol. Genomics* 48 912–921. 10.1152/physiolgenomics.00091.2016 27789736 PMC5206387

[B20] van Waning J. I. Caliskan K. Hoedemaekers Y. M. van Spaendonck-Zwarts K. Y. Baas A. F. Boekholdt S. M. (2018). Genetics, clinical features, and long-term outcome of noncompaction cardiomyopathy. *J. Am. Coll. Cardiol.* 71 711–722. 10.1016/j.jacc.2017.12.019 29447731

[B21] van Waning J. I. Caliskan K. Michels M. Schinkel A. F. L. Hirsch A. Dalinghaus M. (2019a). Cardiac phenotypes, genetics, and risks in familial noncompaction cardiomyopathy. *J. Am. Coll. Cardiol.* 73 1601–1611. 10.1016/j.jacc.2018.12.085 30947911

[B22] van Waning J. I. Moesker J. Heijsman D. Boersma E. Majoor-Krakauer D. (2019b). Systematic review of genotype-phenotype correlations in noncompaction cardiomyopathy. *J. Am. Heart Assoc.* 8:e12993. 10.1161/JAHA.119.012993 31771441 PMC6912966

[B23] Vaz-Drago R. Custódio N. Carmo-Fonseca M. (2017). Deep intronic mutations and human disease. *Hum. Genet.* 136 1093–1111. 10.1007/s00439-017-1809-4 28497172

[B24] Xu X. Jiang H. Lu Y. Zhang M. Cheng C. Xue F. (2019). Deficiency of NONO is associated with impaired cardiac function and fibrosis in mice. *J. Mol. Cell. Cardiol.* 137 46–58. 10.1016/j.yjmcc.2019.10.004 31634484

[B25] Xue Y. Ankala A. Wilcox W. R. Hegde M. R. (2015). Solving the molecular diagnostic testing conundrum for Mendelian disorders in the era of next-generation sequencing: single-gene, gene panel, or exome/genome sequencing. *Genet. Med.* 17 444–451. 10.1038/gim.2014.122 25232854

[B26] Yeo G. Burge C. B. (2004). Maximum entropy modeling of short sequence motifs with applications to RNA splicing signals. *J. Comput. Biol.* 11 377–394. 10.1089/1066527041410418 15285897

